# Targeting Mitochondrial Sirtuins in Age-Related Neurodegenerative Diseases and Fibrosis

**DOI:** 10.14336/AD.2023.0203

**Published:** 2023-10-01

**Authors:** Haoxiang Xiao, Yuqiao Xie, Kaiwen Xi, Jinyi Xie, Mingyue Liu, Yangming Zhang, Zishuo Cheng, Wenting Wang, Baolin Guo, Shengxi Wu

**Affiliations:** ^1^Department of Neurobiology, School of Basic Medicine, Fourth Military Medical University, Xi’an, China.; ^2^Medical School, Yan’an University, Yan’an, China

**Keywords:** aging, sirtuin, fibrosis, neurodegenerative diseases, excessive extracellular matrix

## Abstract

Aging is a natural and complex biological process that is associated with widespread functional declines in numerous physiological processes, terminally affecting multiple organs and tissues. Fibrosis and neurodegenerative diseases (NDs) often occur with aging, imposing large burdens on public health worldwide, and there are currently no effective treatment strategies for these diseases. Mitochondrial sirtuins (SIRT3-5), which are members of the sirtuin family of NAD^+^-dependent deacylases and ADP-ribosyltransferases, are capable of regulating mitochondrial function by modifying mitochondrial proteins that participate in the regulation of cell survival under various physiological and pathological conditions. A growing body of evidence has revealed that SIRT3-5 exert protective effects against fibrosis in multiple organs and tissues, including the heart, liver, and kidney. SIRT3-5 are also involved in multiple age-related NDs, including Alzheimer’s disease, Parkinson’s disease, and Huntington's disease. Furthermore, SIRT3-5 have been noted as promising targets for antifibrotic therapies and the treatment of NDs. This review systematically highlights recent advances in knowledge regarding the role of SIRT3-5 in fibrosis and NDs and discusses SIRT3-5 as therapeutic targets for NDs and fibrosis.

## 1. Introduction

According to *World Population Ageing 2020*, there are 727 million people aged 65 years or older in the world, and the number of people aged 60 years or over worldwide is projected to more than double in the next 28 years, reaching over 1.5 billion [1]. There is no doubt that population aging is a success story, reflecting advancements in public health, medicine, and social development, especially in developed countries. Despite these improvements in life expectancy, aging has arguably become the most dreaded disease in the elderly [2]. Aging is a natural and complex life process that is associated with widespread functional declines in numerous physiological processes, ultimately affecting multiple organs and tissues. However, it remains unknown whether aging has a unified causal mechanism or is a combination of multiple factors. It is worth noting that neurodegenerative diseases (NDs), which are a class of age-related diseases, are a global public health issue that contributes to an enormous burden on society and are one of the main causes of disability in elderly individuals worldwide.

Compelling evidence has indicated that aging is the primary risk factor for most NDs, such as Alzheimer’s disease (AD), Parkinson’s disease (PD), Huntington's disease (HD), and amyotrophic lateral sclerosis (ALS) [3]. Most common NDs feature the accumulation of protein aggregates, some of which are toxic to cells. Amyloid β peptide (Aβ), an aggregative peptide that is prominent in the brain plaques that characterize AD patients, has been shown to have neurotoxic effects [4]. The CAG trinucleotide repeat in the gene encoding the huntingtin protein results in the aberrant accumulation of huntingtin protein, which is the main pathological feature of HD [5]. To date, clinical treatment for NDs has been limited to symptomatic treatment to delay progression, and the results of such treatment have often been unsatisfactory. Fibrosis is the abnormal deposition of extracellular matrix (ECM) and is commonly observed in multiple age-related diseases. Fibrosis is responsible for a heavy burden of disease, and there is still no effective method to prevent or treat it. A great deal of evidence indicates that oxidative stress, cellular metabolism, and autophagy play central roles in the pathogenesis of NDs and fibrosis. The mechanisms underlying these functions involve sirtuins, which can remove a large number of acyl modifications from cellular proteins.

Sirtuins are involved in the metabolic regulation of prokaryotes and eukaryotes as NAD-dependent deacetylases [6]. Over the past two decades, the role of sirtuins in aging and age-related NDs, such as AD, PD, and HD, has been gradually uncovered [7, 8]. Studies have proven that sirtuins play crucial roles in various signaling networks associated with aging and NDs, including nuclear factor-kappa B (NF-κB), AMP-activated protein kinase (AMPK), mammalian/mechanistic target of rapamycin (mTOR), and p53 [9]. These mechanisms are potential targets for preventing or treating age-related dysfunction and could extend a healthy lifespan. Therefore, a better understanding of the neuromolecular basis involved in the aging process will help us identify the specific biochemical pathways of ND and fibrosis, which is very important for developing targeted therapies to promote health and longevity.

**Figure 1.**
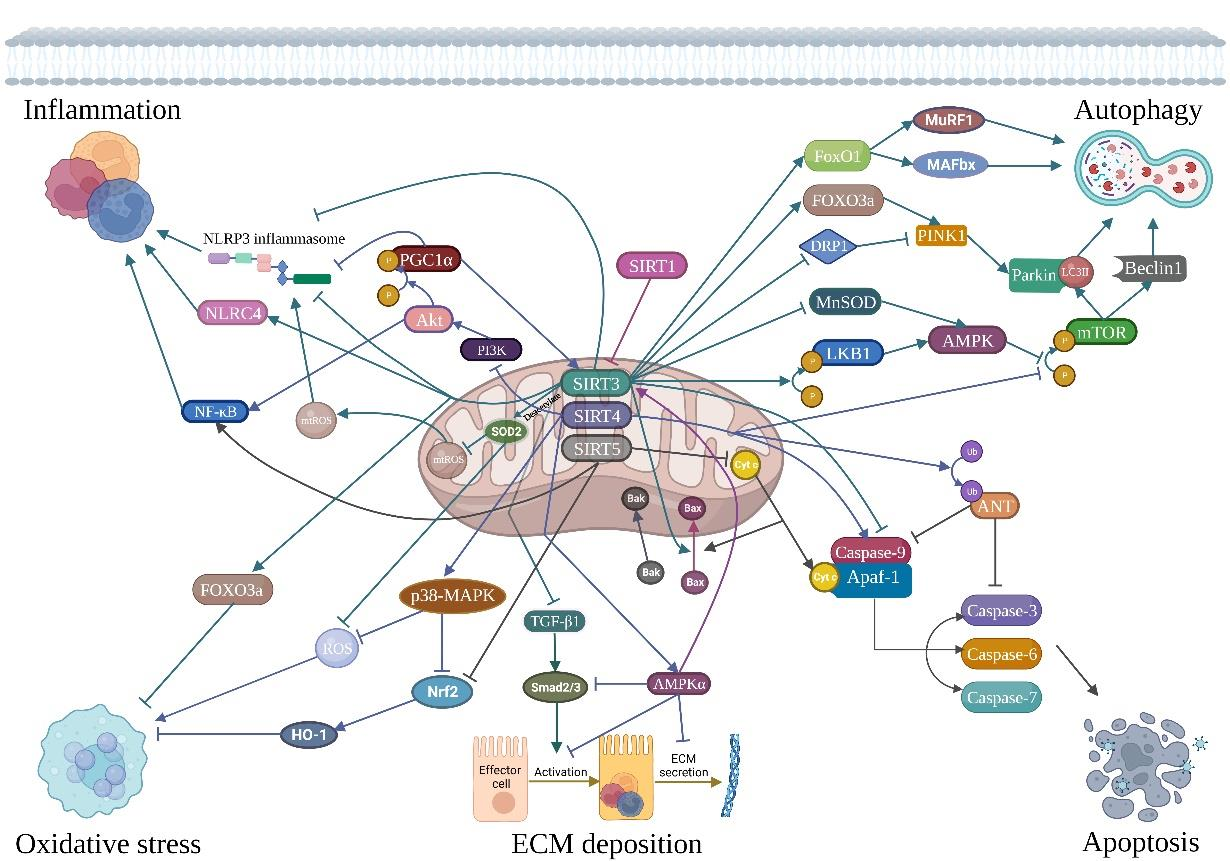
Overview of the biological actions of mitochondrial sirtuins (SIRT3-5). Mitochondrial sirtuins (SIRT3-5), with their unique enzymatic actions, take part in a variety of signaling pathways to regulate inflammation, oxidative stress, autophagy, apoptosis, and inhibit ECM deposition. Compared with SIRT4 and SIRT5, SIRT3 has been extensively studied at present. SIRT3 can participate in inflammation by regulating inflammasome activation and related inflammatory signals such as NF-kB. In addition, due to its unique deacetylase activity, SIRT3 can also regulate a variety of proteins to regulate oxidative stress, autophagy, and apoptosis processes.

In this review, we focus on the roles of sirtuins in age-related NDs and fibrosis, which have been elucidated by recent advances in research. First, we describe the discovery of sirtuins. We then elaborate on the biological actions of sirtuins and their functions in age-related NDs and fibrosis. Finally, we discuss therapeutic targets and possible mechanisms. In conclusion, the information collected here will serve as a comprehensive reference for mitochondrial sirtuin signaling in NDs and fibrosis and may be helpful in designing future experimental studies to increase the potential use of the sirtuin signaling pathway as a therapeutic target in the future (Fig. 1).

## 2. Background of sirtuins

Sirtuins, also known as class III histone deacetylases (HDACs), are homologs of mammalian silencing information regulator 2 (Sir2) that are highly conserved and were first found in the budding yeast *Saccharomyces cerevisiae* [10]. In mammals, the sirtuin family consists of seven members (SIRT1-7), which exhibit diversity in subcellular localization and enzymatic activity [11]. SIRT1, SIRT6, and SIRT7 are localized in the nucleus and take charge of deacetylating histones and regulating gene expression [12]. Notably, SIRT1 was also recently detected in the cytoplasm in certain cell lines, and cytoplasm-localized SIRT1 enhances apoptosis [13]. Although SIRT2 is mainly expressed in the cytoplasm, it can act as a nucleoprotein to regulate the cell cycle [14, 15]. SIRT3, SIRT4, and SIRT5, which are expressed only in mitochondria, are responsible for regulating energy metabolism [16]. In terms of enzyme activity, SIRT1, SIRT2, SIRT3, and SIRT7 exhibit NAD-dependent deacetylase activity [17]. SIRT4 is an ADP-ribosyltransferase and has deacylase as well as substrate-specific deacetylase and lipoamidase activity [18-20]. SIRT5 has NAD-dependent demalonylase, desuccinylase, and deglutarylase activities [21, 22]. SIRT6 was initially found to have deacetylase and ADP-ribosyltransferase activity and has been recently shown to have more robust de-fatty-acylation activity than deacetylation activity [23, 24]. Although these proteins vary in their functions, they take part in some pathological changes and work jointly to maintain homeostasis. In addition, genetic research has divided human sirtuin genes into four categories based on their different localization, activity, and function (Table 1) [25]. Class I sirtuins (SIRT1, SIRT2, SIRT3) have robust NAD^+^-dependent deacetylase activity, while class II sirtuins (SIRT4) show ADP-ribosyltransferase activity [18-20]. Class III sirtuins (SIRT5) have NAD-dependent deformylase and desuccinylase activity in addition to deacetylase activity [21, 22]. Class IV sirtuins (SIRT6 and SIRT7) are fewer active deacetylases than class I sirtuins. SIRT3-5 possess NAD^+^-dependent deacylases and ADP-ribosyltransferases and are mainly localized in mitochondria, where they regulate mitochondrial function by modifying mitochondrial proteins that participate in the regulation of cell survival under various physiological and pathological conditions [26, 27]. The ratio of NAD^+^ to its reduced counterpart NADH is closely related to cell and mitochondrial metabolism. NAD is a cofactor in redox reactions and other signaling pathways. There is convincing evidence that the NAD^+^ levels in multiple organs, such as the brain, decrease with aging [27-29]. Notably, mitochondrial sirtuin activity seems to be regulated by NAD^+^ or the NAD^+^/NADH ratio [30]. For example, the restoration of NAD^+^ levels during senescence may activate mitochondrial sirtuins directly, thus maintaining homeostasis [31]. However, the downstream targets of NAD^+^ or the NAD^+^/NADH ratio and the signaling pathways are still unclear.

**Table1:** Summary of the mammalian sirtuins.

Sirtuin	Location	Enzymatic activity	Class
SIRT1	Nucleus and cytoplasm	NAD-dependent deacetylase	I
SIRT2	Cytoplasm	NAD-dependent deacetylase	I
SIRT3	Mitochondria	NAD-dependent deacetylase	I
SIRT4	Mitochondria	ADP-ribosyltransferase	II
SIRT5	Mitochondria	NAD-dependent demalonylase, desuccincylase and deacetylase activities	III
SIRT6	Nucleus	NAD-dependent deacetylase, ADP-ribosyltransferase and defatty-aclation	IV
SIRT7	Nucleus	NAD-dependent deacetylase	IV

## 3. Biological actions of mitochondrial sirtuins

### 3.1. Regulating cell energy metabolism and maintaining homeostasis

At present, mitochondrial sirtuins have been widely investigated in the context of nutritional stress and cellular energy metabolism, such as fasting and caloric restriction (CR). Evidence is mounting that mitochondrial sirtuins are involved in fatty acid oxidation. For instance, mice lacking SIRT3 (SIRT3^-/-^) exhibit significant metabolic abnormalities during fasting [32, 33]. As a SUMOylated protein in mitochondria, SIRT3 regulates fatty acid oxidation through NAD^+^-dependent deacetylation. SUMOylation, which controls SIRT3 enzyme activity, inhibits SIRT3 catalytic activity and increases mitochondrial acetylation, while SUMOylation-deficient SIRT3 increases mitochondrial protein deacetylation and fatty acid oxidation [32]. SENP1, a SUMO-specific protease, accumulates in mitochondria and is quickly de-SUMOylated during fasting, resulting in SIRT3 activation and the hyperacetylation of mitochondrial proteins, which reduces mitochondrial metabolic adaptation in response to fasting [33]. In addition, hyperacetylated long-chain acyl-coenzyme A dehydrogenase (LCAD) in SIRT3^-/-^ mice has been detected during fasting, which leads to a decrease in its enzyme activity and fatty acid oxidation disorders characterized by decreased ATP levels and intolerance to cold exposure [33]. Notably, SIRT3 can deacetylate LCAD in vitro and in vivo in wild-type (WT) mice under fasting conditions, suggesting that SIRT3 regulates mitochondrial intermediate metabolism and fatty acid utilization during fasting [33]. A previous study identified acetyl-CoA synthetase 2 (AceCS2) as the substrate of SIRT3 [34]. AceCS2 was deactivated after acetylation and rapidly reactivated by SIRT3-mediated deacetylation [35]. Intriguingly, some of the metabolic changes in SIRT3^-/-^ mice were also observed in AceCS2^-/-^ mice [34]. For instance, ATP levels were reduced by 50% in the skeletal muscle of fasted AceCS2^-/-^ mice compared with AceCS2^+/+^ mice, and body temperature was significantly reduced in fasted AceCS2^-/-^ mice [36]. In addition, SIRT4 levels in the liver were decreased, and the expression of liver peroxisome proliferator-activated receptor α (PPAR α) target genes related to fatty acid catabolism was increased during fasting. SIRT4 overexpression reversed these effects, thus reducing the oxidation rate of fatty acids [37]. Notably, knockout (KO) of SIRT4 at the cellular level leads to a significant increase in the gene expression of mitochondrial and fatty acid metabolizing enzymes in hepatocytes and increases SIRT1 mRNA and protein levels, suggesting that SIRT4 regulates lipid metabolism in a SIRT1-dependent manner [38]. Further studies showed that the SIRT4-AMPK-SIRT1 pathway can affect the lipid metabolism of cells. Furthermore, SIRT4 can deacetylate and inhibit malonyl coenzyme A decarboxylase (MCD). Mice lacking SIRT4 show increased MCD activity and decreased malonyl coenzyme levels in skeletal muscle and white adipose tissue. SIRT4-KO mice exhibit lipid metabolism disorders, which lead to increased exercise tolerance [39]. In summary, SIRT3 and SIRT4 seem to play opposite roles in regulating fatty acid oxidation. Increased expression of SIRT3 during fasting can accelerate fatty acid oxidation to overcome adversity, while overexpression of SIRT4 can reduce the rate of fatty acid oxidation. Whether there is an interaction between SIRT3 and SIRT4 and the relationship between them are still unclear.

SIRT3 also participates in protein metabolism. SIRT3^-/-^ mice display perturbations in the levels of numerous acylcarnitines and urea cycle metabolites [40]. SIRT3 directly regulates ornithine transcarbamoylase activity and promotes the urea cycle during CR, suggesting that SIRT3 promotes amino acid catabolism and β-oxidation to modulate mitochondria during low energy input [40]. Studies have shown that a lack of SIRT3 activity leads to brain glucose metabolism disorders [41]. Palmitoleic acid reduces gluconeogenesis by downregulating SIRT3 expression and reducing gluconeogenic enzyme activity in animals receiving a high-fat diet, indicating the involvement of SIRT3 in glycometabolism [42]. Notably, SIRT4 plays a role in the mitochondria of pancreatic beta cells by inhibiting the activity of glutamate dehydrogenase (GDH) through ADP ribosylation, thus downregulating insulin secretion in response to amino acids [43, 44]. SIRT4 is also involved in controlling leucine metabolism and insulin secretion as a lysine deacylase [19]. In addition, SIRT4 inhibits tumors by inhibiting the metabolism of glutamine in the tricarboxylic acid (TCA) cycle [45].

SIRT5 is widely expressed in the brain, heart, liver, and skeletal muscle and is an effective lysine desuccinylase and demalonylase in vitro. Among mitochondrial sirtuins, SIRT5 has rarely been studied, and SIRT5 deficiency does not lead to any obvious metabolic abnormalities under normal or high-fat diet conditions [46]. Recent studies have shown that SIRT5 depletion enhances glutamine and glutathione metabolism and promotes tumorigenesis through acetylation-mediated glutamate oxaloacetate transaminase 1 (GOT1) activation, while the SIRT5-selective activator MC3138 inhibits the proliferation of tumor cells [47]. Furthermore, the deletion of SIRT5 results in the accumulation of long-chain acylcarnitine and a reduction in β-hydroxybutyrate production in vivo by regulating the succinylation of the rate-limiting ketogenic enzyme 3-hydroxy-3-methyl-glutaryl coenzyme a synthase 2 (HMGCS2), suggesting that SIRT5 is implicated in ketone production [21]. SIRT5 also mediates the desuccinylation of p53 at lysine 120 (K120), inhibits the activation of p53, and plays an important role in DNA damage. SIRT5 plays an important role in the regulation of cardiac function. ECHA, a protein involved in the oxidation of fatty acids, is a major enzyme regulated by SIRT5 and affects heart function. Under fasting conditions, the ECHA activity of SIRT5-KO mice was reduced, the abundance of long-chain acyl-coenzyme A was increased, and the cardiac ATP level was decreased [48]. In addition, SIRT5 can modulate other metabolic pathways in mice, such as glycolysis, the TCA cycle, and amino acid metabolism, but its correlates molecules and specific targets are still unknown.

Overall, SIRT3-5 can regulate the dynamic switching of various metabolic pathways between different substrates and mediate the treatment of by-products in the metabolic process. SIRT3-5 deletions combined with nutritional stress and subsequent disorders of glucose, fatty acid and protein metabolism are crucial to the pathological progress related to aging. Meanwhile, the sirtuin family members like SIRT4 and SIRT1 interact with each other thus forming SIRT4-AMPK-SIRT1 pathway to mediate cellular metabolism, but there are limited researches about connections between other different sirtuins, which still needs further exploration. Future research on the upstream regulators of SIRT3-5 and other target molecules involved in these metabolic processes will deepen our understanding of mitochondrial metabolism.

### 3.2. Regulation of inflammatory signaling pathways

Inflammation is the basis of various physiological and pathological processes and occurs in multiple tissues and organs. An increasing amount of compelling evidence claims that mitochondrial sirtuins play important roles in inflammation. Inflammasomes are multimolecular complexes that are well known for their ability to regulate the proteolytic maturation of interleukin (IL)-1β and IL-18 by controlling the activation of the proteolytic enzyme caspase-1 [49]. Several different inflammasomes have been identified, each distinguished by a unique activator, nucleotide-binding domains, leucine-rich-repeat-containing (NLR)/AIM2-like receptor (ALR) family members, and caspase effectors [50]. The NLRP3 inflammasome is a unique innate immune sensor that can be activated by a variety of endogenous metabolic signals, thus inducing inflammation. NLRP3 activation requires two signals, which have been detailed in our previous article [51]. SIRT3 deficient macrophages and WT macrophages have similar ability to stimulate IL-6 production by NLRP3 inflammasome after ATP or melanomycin treatment, which indicates that SIRT3 is indispensable for activation of NLRP3 inflammasome [52]. A significant reduction in SIRT3 expression in the lungs of LPS-induced acute lung injury (ALI) mice was associated with a proinflammatory phenotype characterized by NLRP3 inflammasome activation. Activation of SIRT3 can effectively reduce the activation of NLRP3 and the production of proinflammatory cytokines, including TNF-α, MIP-2, IL-6, IL-1β, and HMGB1, but this protective effect was eliminated in SIRT3^-/-^ mice [53]. Similarly, in mice with streptozotocin (STZ, 60 mg/kg, ip)-induced diabetes mellitus, SIRT3 deletion resulted in the upregulation of inflammatory mediators such as NLRP3, caspase-1 p20, and IL-1 β, ultimately exacerbating diabetic cardiomyopathy [54]. SIRT3 and peroxisome proliferator-activated receptor-γ coactivator 1α (PGC-1α) are considered negative regulators of NLRP3. Krill oil inhibits NLRP3 inflammasome activation by upregulating the expression of SIRT3 and PGC-1α and prevents the pathological injuries associated with diabetic cardiomyopathy [55]. In addition, vitamin D3 attenuates nitrogen mustard-induced cutaneous inflammation by inhibiting the NLRP3 inflammasome through the SIRT3-superoxide dismutase 2 (SOD2)-mitochondrial reactive oxygen species (mtROS) signaling pathway [56]. Another study corroborated that conclusion [57]. Additionally, prolonged fasting inhibits the assembly and activation of the NLRP3 inflammasome through SIRT3-mediated mitochondrial SOD2 deacetylation. In contrast, siRNA knockdown of SIRT3 or SOD2 increases the formation and activation of the NLRP3 inflammasome. Posttranslational modification of NLRC4, another inflammasome inducer, is essential for its activation. SIRT3-mediated deacetylation of NLRC4 at Lys71 or Lys272 could promote its activation [52].

Endothelial inflammation is the most common feature of endothelial dysfunction. LPS decreased the expression of SIRT4 and increased the expression of proinflammatory cytokines (IL-1 β IL-6 and IL-8), the COX-prostaglandin system (COX-2), the ECM remolding enzyme MMP-9 and the adhesion molecule ICAM-1 in human umbilical vein endothelial cells. SIRT4 exerts an anti-inflammatory effect by preventing nuclear translocation of NF-κB and interfering with the NF-κB signaling pathway [58]. Infiltrating regulatory T (Treg) cells are potent anti-inflammatory cells that inhibit neuroinflammation after spinal cord injury (SCI). SIRT4 suppresses the anti-neuroinflammatory activity of Treg cells in SCI through two pathways. First, SIRT4 overexpression blocks the formation of Treg cells from conventional T cells in vitro. Furthermore, SIRT4 downregulates AMPK signaling in Treg cells [59]. In addition, SIRT4 overexpression inhibited the phosphatidylinositol 3-kinase (PI3K)/protein kinase B (Akt)/NF-κB signaling pathway and downregulated the expression of inflammatory cytokines in oxidized low-density lipoprotein-induced human umbilical vein endothelial cells [60]. SIRT5 desuccinylates and activates pyruvate kinase M2 to block macrophage IL-1β production and prevent DSS-induced colitis in mice [61]. In addition, SIRT5 promotes the acetylation of p65 and the activation of the NF-κB pathway, as well as its downstream cytokines, and enhances the innate inflammatory responses in normal and endotoxin-tolerant macrophages [62]. Numerous studies have shown that pro-inflammatory cytokines, such as IL-1β and TNF-α, are abnormally elevated in NDs [63, 64]. In a PD model, mitochondrial sirtuins alleviate inflammation by inhibiting the NF-κB signaling pathway, thus reducing DA neuron damage [62, 65, 66].

Glial cells are intrinsic components of the central nervous system that play a special role in neuroinflammation and neurotoxicity. Their dysfunction is also an important driver of neurodegenerative diseases such as AD and HD [67]. As the sirtuin family is implicated in neuroinflammation, the role of crosstalk between these proteins and glial cells in mediating inflammation has received growing attention. Deacetylation of the NF-κB protein complex by SIRT1 can repress NF-κB expression, thus decreasing the levels of pro-inflammatory cytokines secreted due to microglial activation in the context of LPS administration [68]. An increase in the number of microglia during the later stage of neurodegeneration can be observed as a biomarker in AD with increasing SIRT5, whose function contrasts with the neuroprotective functions of SIRT1 and SIRT3 [69]. Differential expression of the SIRT family in astrocytes as well as microglia can be induced by psychostimulants and opioids, which may interfere with the inflammatory process in the central nervous system, but this topic has not been extensively studied [70]. In addition, interactions between glial cells and sirtuins may correlate with cellular processes and energy metabolism, thus contributing to the aging process in the brain. Overall, the roles of SIRT4 and SIRT5 in inflammation are relatively poorly understood, and subsequent studies should be conducted to delineate the complete anti-inflammatory pathways of mitochondrial sirtuins.

### 3.3. Inhibiting oxidative stress

Oxidative stress, which causes DNA damage and increases the oxidation of lipids and proteins, is an important feature of aging and neurodegeneration. The imbalance in the generation of reactive oxygen species (ROS) and the antioxidant defense system accounts for oxidative stress. Compelling evidence has linked mitochondrial sirtuins to oxidative stress. Numerous studies have shown that SIRT3 has multiple antioxidant functions in mitochondria. SIRT3 regulates the ROS balance through three main pathways. First and most commonly, SIRT3 directly interacts with and deacetylates SOD2, which enhances SOD2 activity and promotes ROS clearance [71]. For example, SIRT3 depletion in SIRT3^-/-^ mice results in oxidative stress, endothelial dysfunction, and hypertension due to the hyperacetylation of mitochondrial SOD2, whereas increasing SIRT3 expression reverses these effects [72, 73]. Mitochondria are the main source of cellular ROS; approximately 90% of cellular ROS are produced in mitochondria. This is the second pathway by which SIRT3 promotes the function of the electron transport chain and reduces oxidative stress [74]. SIRT3-mediated deacetylation is involved in the regulation of mitochondrial ROS, which can be produced at complex I, II, and III sites. Notably, the enzymatic activity of mitochondrial complexes I, II, and III is suppressed in the brains of PD patients, which leads to excessive ROS production [75-77]. Encouragingly, upregulation of SIRT3 significantly reduced ROS production and ameliorated neuronal degeneration [78]. In addition, SIRT3 regulates mitochondrial ROS levels by modulating the subunits of the TCA cycle enzymes pyruvate dehydrogenase and aconitase, both of which are related to increased mitochondrial ROS production [74, 79]. Studies have shown that ND2 and ND4 gene expression levels are significantly reduced in AD patients, which leads to elevated ROS levels [80]. SIRT3 reduces mitochondrial ROS accumulation and alleviates neuronal damage through the p53-ND2/ND4 pathway based on its deacetylation activity [80]. PGC-1α is a key regulator of mitochondrial biogenesis and metabolism. It was first found in brown adipose tissue and has been shown to be highly expressed in highly oxidized cells such as neurons and cardiomyocytes [81, 82]. Interestingly, both Akt and PGC-1α are upstream regulators of SIRT3, and Akt can directly phosphorylate PGC-1α and inhibit PGC-1α recruitment to its homologous promoter region [83]. Additionally, mTOR is an upstream signal of PGC-1α that leads to increased expression of SIRT3 and metabolic genes, increases NADPH levels and the proportion of oxidized glutathione, and protects cells from oxidative damage by upregulating PGC-1α expression [84].

Notably, the SIRT1-SIRT3 axis plays a pivotal role in oxidative stress. SIRT1 silencing was shown to increase SIRT3 promoter activity, and this effect was dependent on the presence of SP1 and ZF5 recognition sequences on the SIRT3 promoter. Mechanistically, SIRT1 inhibits the activity of SIRT3 by binding to and deacetylating the transcription inhibitor ZF5 [85]. Another study demonstrated that the 5-ALA-PDT-induced reduction in SIRT1 protein levels promoted SIRT3 expression, increased SOD2 activity and decreased mitochondrial ROS production [86]. In addition, activation of the SIRT1-SIRT3-FOXO3a pathway can protect neurons from oxidative stress [87]. Notably, the effects of the SIRT1-SIRT3 axis on oxidative stress occur only when AMPK is activated [88]. An AMPKα deficiency study identified that AMPKα was responsible for the increases in SIRT3 expression in vitro and in vivo [89]. This leads us to consider whether SIRT1 inhibition would necessarily increase SIRT3 expression. Based on current research, the answer is still unclear.

In recent years, the roles of SIRT4 and SIRT5 in oxidative stress have been gradually revealed. Heme oxygenase-1 (HO-1) is part of an endogenous defense system against oxidative stress. SIRT4 modulates ROS and HO-1 expression by accommodating p38-MAPK phosphorylation [90]. SIRT5 deletion increases oxidative stress, including hydrogen peroxide production and oxidative DNA damage [91]. In mitochondria, SIRT5 fights oxidative stress mainly through its desuccinylation and desuccinylation. For example, SIRT5 can inhibit acyl-CoA oxidase 1 activity by inhibiting the formation of the ACOX1 active dimer through desuccinylation in vivo or vitro, ultimately inhibiting peroxisome-induced oxidative stress [91]. In addition, SIRT5 plays an antioxidant role through maintaining NADPH homeostasis. NADPH is the main intracellular reductant and keeps glutathione in its reduced form GSH, which can clear ROS and protect cells from oxidative damage. SIRT5 desuccinylates and deglutarylates isocitrate dehydrogenase 2 and glucose-6-phosphate dehydro-genase, respectively, thereby activating these two NADPH-producing enzymes, increasing GSH and enhancing the ability to scavenge ROS [92]. Succinate metabolic disorders in myocardial tissue during ischemia-reperfusion can lead to myocardial oxidative injury. SIRT5 can desuccinylate and inactivate succinate dehydrogenase, thereby reducing ROS-induced oxidative damage [93]. SIRT5 inhibits PKM 2 activity through desuccinylation of PKM 2 K498 and plays an antioxidant role [94]. SIRT5 can also regulate oxidative stress by regulating the Nrf2/HO-1 signaling pathway [95].

### 3.4. Bidirectional regulation of apoptosis

Apoptosis is a complex but highly precise process of programmed cell death. It is clear that the traditional apoptotic pathways include the mitochondrial-mediated intrinsic apoptotic pathway and death receptor (DR)-dependent exogenous pathway [96]. Caspases are key molecules in all apoptotic pathways. The exogenous pathway is mediated by DRs, which initiate the proapoptotic cascade of caspases. In the intrinsic apoptotic pathway, multiple stimuli contribute to the increase in mitochondrial membrane permeability, which leads to the translocation of cytochrome c (cyt c) to the cytoplasm. In the cytoplasm, cyt c binds with apoptotic protease activator 1 (Apaf-1) and caspase-9 to form a large complex called the apoptosome. Subsequently, downstream caspases such as caspase-3, caspase-6, and caspase-7 are activated, leading to apoptosis.

The pro-apoptotic proteins Bax and Bak are usually located in the cytoplasm, but when mitochondrial apoptosis occurs, they translocate to the outer membrane of mitochondria and form permeable transition pores that allow the release of apoptotic factors to induce mitochondrial rupture. In contrast, anti-apoptosis proteins Bcl-2 and Bcl-xL inhibit the activation of Bax and Bak and maintain mitochondrial integrity [97].

Recently, the role of SIRT3 in apoptosis has been gradually revealed. SIRT3 inhibits apoptosis in a variety of disease models. In a rat osteoarthritis model, the activation of Bax and caspase 3/9 and the downregulation of Bcl-2, which were induced by IL-1β in SIRT3-overexpressing chondrocytes, were significantly reduced, indicating that SIRT3 protected chondrocytes from IL-1β-induced apoptosis [98]. A previous study reported that peroxiredoxin 3 (PRDX3), a member of the thiol peroxidase family that is localized in mitochondria, effectively inhibited apoptosis [99]. SIRT3 can reduce mitochondrial apoptosis by deacetylating PRDX3 at K253 and decreasing the level and activity of cleaved caspase-3 during intestinal ischemia-reperfusion [100]. However, little is known about how PRDX3 affects the apoptosis pathway. In addition, SIRT3 can deacetylate Ku70 and enhance the Ku70-Bax interaction, thus blocking the transfer of Bax to mitochondria to protect cells from apoptosis [101]. Notably, SIRT3 has also been reported to be involved in promoting apoptosis. SIRT3 overexpression increased mitochondrial Bax levels and promoted the apoptosis signaling pathway. However, GSK-3 β inhibitors blocked this effect, suggesting that SIRT3 may regulate the GSK-3β/Bax axis and affect apoptosis [102]. In addition, SIRT3 also plays a proapoptotic role in a range of human cancer cell lines derived from colorectal cancers and osteosarcoma [103].

SIRT4 is downregulated at both the transcript and protein levels in cardiomyocytes after myocardial ischemia-reperfusion (MI-R) [104]. Intriguingly, SIRT4 reduced cardiomyocyte apoptosis by decreasing the protein level of cleaved caspase-3 (cl-cas3) after MI-R in vivo, indicating that SIRT4 could reduce MI-R injury by inhibiting apoptosis [104]. In addition, SIRT4 overexpression significantly increased apoptosis in BCPAP cells (a human thyroid cancer cell line), which was characterized by the increased expression of caspase 318 KD and caspase 9 and the decreased expression of p65 [105]. P21-activated kinase 6 (PAK6) is a member of the class II Pak family, which is mainly located in the inner membrane of mitochondria and can promote ubiquitin-mediated proteolysis of SIRT4 [106]. Adenine nucleotide translocase (ANT) is a mitochondrial protein that inhibits caspase 3 and 9 and inhibits apoptosis in prostate cancer [106]. A study revealed that SIRT4 could deacetylate ANT2 at K105 and promote its ubiquitination-mediated degradation, while PAK6 downregulated SIRT4 and reversed these effects. In addition, PAK6 could directly phosphorylate ANT2 at T107 to inhibit apoptosis in prostate cancer cells [106]. Cyt c transport from mitochondria to the cytoplasm is a key step in the initiation and/or progression of apoptosis. SIRT5 deacetylates cyt c and decreases the release of cyt c from mitochondria, which upregulates the proapoptotic proteins cl-cas3, cl-PARP, and Bax and downregulates Bcl-2, ultimately inhibiting apoptosis in hepatocellular carcinoma cells [107].

### 3.5. Regulation of autophagy and mitochondrial autophagy

Autophagy is a process in which cells clear aged and damaged organelles and proteins through lysosomes and is considered to be a key player in cellular and organismal metabolism [108]. Dysregulation of autophagy contributes to a variety of diseases, including obesity, diabetes, and atherosclerosis [109]. Numerous studies have detected that SIRT3 is involved in regulating autophagy in various pathological processes. PPARα is a key regulator of mitochondrial homeostasis and autophagy activation during infection. SIRT3 activates autophagy to promote antimicrobial host defense during *Mycobacterium tuberculosis* infection via the SIRT3-PPARA axis [110]. SIRT3 overexpression also promotes liver kinase B1 (LKB1) phosphorylation, subsequently activating AMPK and reducing mTOR phosphorylation, which ultimately increases the levels of the autophagy markers LC3II (microtubule associated protein 1 light chain 3-II) and Beclin1 (the mammalian ortholog of yeast ATG6) [111]. The autophagy inhibitor 3-MA weakened the protective effect of SIRT3, suggesting that the SIRT3-LKB1-AMPK pathway could partially activate autophagy. Notably, in the livers of mice exposed to a saturated fatty acid-rich high-fat diet, SIRT3 overexpression caused manganese superoxide dismutase (MnSOD) deacetylation and further led to AMPK inhibition and mTORC1 activation, resulting in autophagy suppression [112]. Melatonin treatment suppressed cadmium-induced autophagy by increasing the activity of SIRT3 but not its expression, further promoting SOD2 deacetylation and inhibiting mtROS production [113]. These results suggest that SIRT3 may play opposite roles in different pathological processes through different signaling pathways.

In addition, SIRT3 participates in cardiac protection by regulating autophagy. Current evidence suggests that forkhead box O (FoxO) transcription factors play multiple roles in autophagy regulation and disorders. SIRT3 can bind with FoxO1 in the cytoplasm to promote its deacetylation. Subsequently, deacetylated FoxO1 translocates to the nucleus and promotes downstream E3 ubiquitin ligases, such as muscle ring finger 1 (MuRF1) and muscle atrophy F-box (MAFbx, atrogin1), promoting autophagy and mitigating cardiomyocyte hypertrophy [114]. Notably, there may be positive feedback between SIRT3 and FoxO1. In the context of FoxO1 gene KO, the expression of SIRT3 was also downregulated to a considerable extent. However, the manner in which FoxO1 regulates SIRT3 and the effect of this regulation merit further study. Similarly, SIRT3 improves cardiac dysfunction caused by diabetes by activating FoxO3a-mediated mitochondrial autophagy [115]. Mechanistically, PINK1 (PTEN-induced putative kinase 1) is activated by FoxO3a and upregulates the expression of Parkin, which is a multifunctional E3 ubiquitin ligase that can mediate the interaction of the autophagy junction protein p62/sqstm1/sequestosome-1 with disordered mitochondria and with LC3-II on autophagosomes, leading to the activation of mitochondrial division and phagocytosis [116]. In addition, spinacetin protects against doxorubicin-induced cardiotoxicity in vitro and in vivo by initiating protective autophagy through the SIRT3/AMPK/mTOR pathway [117]. Notably, this pathway also plays a protective role in sepsis-induced acute kidney injury (AKI) by inducing autophagy [118]. Dynamin-related protein 1 (DRP1) is recruited to mitochondria during mitochondrial dysfunction and participates in autophagic clearance of damaged mitochondria. SIRT3 negatively regulates the expression of DRP1 and increases the expression of PINK1, Parkin and BNIP3, thereby increasing the level of autophagy and protecting the kidney from ischemia-reperfusion injury [119]. SIRT3 can also ameliorate osteoarthritis by regulating chondrocyte autophagy by inhibiting the IL-1β-induced PI3K/Akt/mTOR signaling pathway [98].

SIRT4 overexpression alleviates adriamycin-induced cardiotoxicity by inhibiting Akt/mTOR-dependent autophagy [120]. SIRT4 may have weak enzyme activity, and little research has been reported on its role in autophagy. SIRT5 in non-liver cells regulates ammonia-induced autophagy by promoting the desuccinylation of glutaminase and regulating glutamine metabolism and ammonia production [121]. Notably, SIRT5 accelerates the growth of colorectal cancer cells by inducing autophagy by deacetylating lactate dehydrogenase B at lysine-329 [122]. In addition, autophagy and SIRT5 expression are impaired in an AD mouse model [64]. SIRT5 overexpression notably suppresses oxidative stress by activating autophagy. However, the mechanism by which SIRT5 activates autophagy and further inhibits oxidative stress remains unclear. Based on these results, mitochondrial sirtuins, especially SIRT3, are double-edged swords that regulate autophagy. In the future, we should focus on the different conditions that lead to these opposing effects and how to regulate mitochondrial sirtuins to make them play a protective role in different pathological processes.

### 3.6. Inhibition of extracellular matrix deposition

The extracellular matrix (ECM) acts as a dynamic regulator of body systems by taking part in many different communications between organs and tissue cells, including cell proliferation and survival, migration, differentiation, autophagy, and immunity modulation. The building blocks of ECM include collagens, proteoglycans and glycosaminoglycans, elastin and elastic fibers, laminins, fibronectin, and other proteins/glycoproteins [123]. Changes in this intricate network can contribute to many human diseases, such as cancer, diabetes, and organ fibrosis. Fibrosis is one of the most extensively studied ECM-induced diseases [124], and since sirtuins are among the factors necessary to prevent fibrosis, the pathological process of fibrosis may be intertwined with the ECM and sirtuins.

Fibrosis is a dynamic reaction composed of three continuous processes: primary inflammatory response, effector cell activation and ECM secretion. Transforming growth factor-β1 (TGF-β1) is a key cytokine that mediates the transformation of fibroblasts into myofibroblasts, which can synthesize ECM. Notably, SIRT3 deacetylates and activates glycogen synthase kinase 3β (GSK3β), a signaling kinase that interferes with TGF-β signaling, thus inhibiting TGF-β1-mediated fibroblast to myofibroblast transformation and inhibiting the deposition of ECM [125]. AMPK is another key regulator of the process of fibrosis and has been shown to play a protective role in a variety of fibrotic diseases [89, 126]. SIRT4 has been shown to activate AMPKα, inhibit fibroblast proliferation and ECM accumulation, and inhibit the TGF-β/Smad2/3 signaling pathways [127, 128]. In addition, AMPK plays an antifibrotic role by activating SIRT3 [129, 130]. In conclusion, according to current studies, mitochondrial sirtuins mainly inhibit ECM deposition through the TGF-β/AMPK pathway, thus playing an antifibrotic role.

## 4. Mitochondrial sirtuins in neurodegenerative diseases

**Figure 2.**
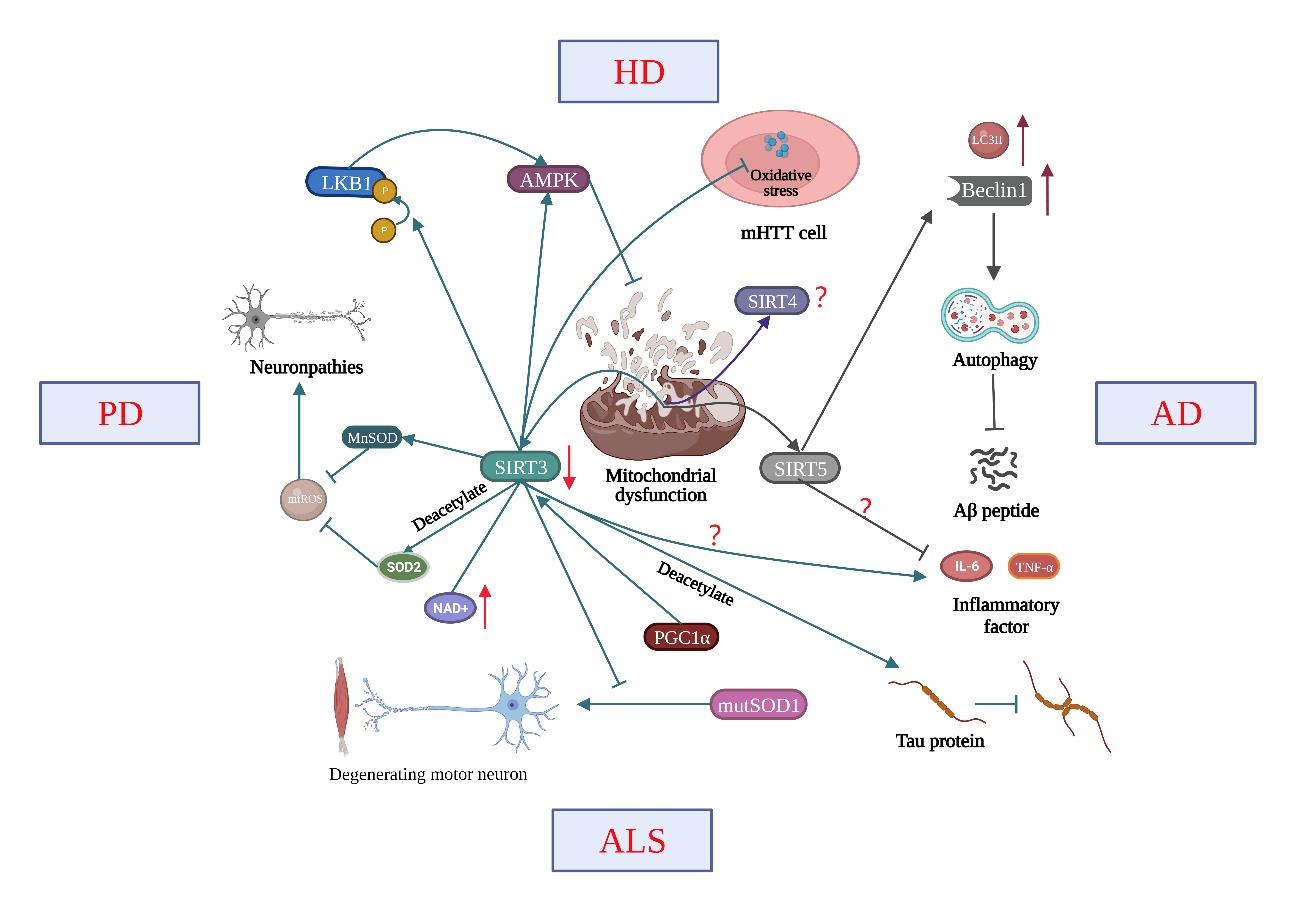
Mitochondrial sirtuins (SIRT3-5) in neurodegenerative diseases. A large number of studies have confirmed that mitochondrial dysfunction is the common pathogenic mechanism of NDs. SIRT3 has been shown to decrease under these disease conditions. It further increases the acetylation level of various proteins, such as Tau protein and SOD2, thus promoting the accumulation of Tau protein and the degeneration of dopaminergic neurons. Compared with SIRT3, the expression changes of SIRT4 and SIRT5 in NDs are still controversial. Notably, studies have shown that SIRT5 can inhibit inflammation and apoptosis and play a protective role in NDs.

A growing body of studies has confirmed that mitochondria play a crucial role in NDs. The role of mitochondrial sirtuins in NDs is summarized below (Fig. 2).

### 4.1. Alzheimer’s disease

AD is an irreversible ND characterized by the accumulation of Aβ peptide and tau protein abnormalities that impair mental ability and interrupt neurocognitive function [131]. In 2019, Alzheimer’s Disease International estimated that there were over 50 million people living with dementia worldwide, which is expected to increase to 152 million by 2050, and two-thirds live in low-income and middle-income countries [132]. Notably, 2/3 of people still think that dementia is a normal stage of aging, rather than an ND, even though in some countries, it is the leading cause of death [132]. More worryingly, a staggering 62% of medical practitioners believe dementia is a normal part of aging [132]. It is notoriously difficult to develop therapeutics for AD due to its complex and unknown pathogenesis and the limitation of the blood-brain barrier. In recent years, with further molecular mechanism research, serum biomarkers have attracted extensive attention and are expected to become targets for the diagnosis and treatment of AD [133].

Compelling evidence has indicated that oxidative stress, mitophagy, and mitochondrial dysfunction play vital roles in the pathologic process of AD [134-137]. Mitochondrial sirtuins are involved in mitochondrial dysfunction and AD pathogenesis [80, 138, 139]. SIRT3 is an important mitochondrial protein that regulates mitochondrial bioactivity. Studies have shown that SIRT3 mRNA is downregulated in the brain tissues, including the entorhinal cortex, the middle temporal gyrus, and the superior frontal gyrus, of AD patients compared to cognitively normal controls, which might promote the progression of AD [140, 141]. Moreover, higher levels of SIRT3 in postmortem human brains are associated with better cognitive performance, including global cognitive ability, naming, verbal learning, and memory [141]. Mechanistically, SIRT3 plays protective roles in AD by regulating Aβ and tau protein. Neuropathological studies of AD have shown a strong association between tau deposits and decreased cognitive function [142]. Notably, SIRT3 can promote the deacetylation of tau at the posttranslational level, inhibiting pathological tau aggregation, which might be a new disease-modifying strategy for drug discovery and biomarker development in AD [141]. As the major genetic risk factor for AD, apolipoprotein E4 (ApoE4) has been shown to reduce ATP production by regulating the PGC-1α-SIRT3 signaling pathway and triggering subsequent mitochondrial oxidative stress and synapse damage, ultimately causing cognitive impairment [143]. Notably, mitochondrially targeted p53 (mito-p53) could reduce mitochondrial DNA-encoded ND2 and ND4 gene expression, resulting in increased ROS levels, while SIRT3 overexpression restored the expression of ND2 and ND4 and improved mitochondrial oxygen consumption by repressing mito-p53 activity in patients with AD [80]. These two studies suggested that SIRT3 protected neurons from oxidative stress damage in AD. In addition, SIRT3 attenuated Aβ-induced neuronal hypometabolism in AD [144]. A recent study linked SIRT3 deficiency to neuroinflammation, which is characterized by elevated expression of IL-1β, TNF-α, and Cox-2 in SIRT3-/- mice at 8 months of age with amyloid pathology and metabolic syndrome [145]. Considering the anti-inflammatory activity of SIRT3, whether SIRT3 overexpression can inhibit neuroinflammation in AD is worthy of further investigation.

SIRT5, a mitochondrial sirtuin with very weak deacetylase activity, has been identified as being involved in the neuropathological process of AD [64]. Interestingly, in contrast to that of SIRT3, increased expression of SIRT5 in human brain tissue during the progression of AD has been detected [69]. However, another study showed downregulated mRNA levels of SIRT5 in an AD mouse model [64]. Different tissue sources and observational parameters may explain these contradictory results. In an APP695/PS1dE9 transgenic mouse model of AD, the overexpression of SIRT5 promoted autophagy, which was characterized by increases in Becn1 and the ratio of LC3b-II/I, as well as suppressed oxidative stress, ultimately clearing Aβ [64]. Moreover, an enhancement in the learning and memory ability of mice, as tested by the Morris water maze, was demonstrated in response to SIRT5 treatment. Previous studies have shown that the number of microglia is increased in the brains of AD patients [146]. Wu *et al.* demonstrated that SIRT5 ameliorated neuronal damage by suppressing the activation of astrocytes and microglia [64]. In addition, SIRT5 overexpression suppressed the AD-mediated increase in the expression of the inflammatory cytokines TNF-α and IL-6, suggesting that SIRT5 overexpression alleviates inflammation and may promote neural functions in AD brains [64]. Unlike SIRT3 and SIRT5, SIRT4 still has an unclear correlation with AD. Although many studies have linked SIRT4 to ND, the specific target of its interaction remains unclear. Intriguingly, SIRT4 also plays a pivotal role in the morphology and function of radial glia in the brain [147]. Whether SIRT4 also regulates glial cells through a similar effect as SIRT5, thus participating in the pathological process of AD, should be further studied. Notably, while the therapies of AD targeting at SIRT3 and SIRT5 is still limited and clinical data is far to reach, the strong SIRT1 activating compound resveratrol has enter into clinical trials for AD but regretfully without exciting results[148].

### 4.2. Parkinson’s disease

PD, which is the second most common progressive neurodegenerative disorder affecting older American adults, as well as the second most common devastating neurodegenerative disorder, is predicted to increase in prevalence as the American population ages [149]. PD is the result of the joint effects of genetic and environmental factors and is characterized by degeneration of the substantia nigra (SN) pars compacta and Lewy body α-synuclein accumulation [150]. Although the replacement of lost dopamine and exercise therapy is commonly used in clinics, PD diagnosis and treatment remain challenging.

Substantial evidence indicates that mitochondrial dysfunction is one of the central pathogenic mechanisms of dopaminergic neuronal degeneration and PD pathogenesis. Dynamic acetylation of mitochondrial proteins plays an important role in maintaining mitochondrial function. Furthermore, the deacetylation of mitochondrial proteins is mainly performed by SIRT3 [138]. Notably, neurons treated with phenyl-1,2,3,6-tetrahydropyridine (MPTP) decreased the expression of SIRT3 protein, which is related to the high acetylation of SOD2 at K130 and ATP synthase β at K485, as well as the loss of dopaminergic neurons. Mogroside V (MV), which is extracted from *Siraitia grosvenorii*, can reduce ROS levels, reverse abnormal MMP, and decrease apoptotic cells through the SIRT3-mediated pathway, thus alleviating motor impairments and dopaminergic neuronal injury in the SN in a PD model [151]. 1-Methyl-4-phenylpyridinium iodide (MPP^+^), an MPTP metabolite, was applied to SH-SY5Y cells to generate a cellular model of PD [151]. MPP^+^ treatment resulted in decreased deacetylation of citrate synthase and isocitrate dehydrogenase 2 by reducing SIRT3 expression and ultimately decreased the enzyme activity of these proteins [152]. SIRT3 inhibits dopaminergic neuronal degeneration in PD by increasing the ROS elimination activity of SOD2 through deacetylation at the K68 site [153]. The age-dependent increase in mitochondrial oxidative stress is widely considered to be a major factor in the loss of dopaminergic neurons in the substantia nigra compacta (SNc) of patients with PD. A study showed that SIRT3 deletion increased oxidative stress and decreased mitochondrial membrane potential in SNc dopaminergic neurons due to increased acetylation and decreased MnSOD activity [153]. SIRT5 has also been suggested to protect against motor deficits and dopaminergic neuron degeneration in MPTP-treated mice [154]. However, it is largely unknown whether and how SIRT5 protects dopaminergic neurons via its deacetylase activity.

### 4.3. Huntington's disease

HD, which is characterized by cognitive dysfunction and the loss of coordination and motor functions, is a progressive, fatal neurodegenerative disorder. HD is caused by a genetically amplified CAG repeat, which encodes an abnormal polyglutamine in the Huntington protein (Htt) [5] At the cellular level, mutant Htt (mHtt) leads to neuronal dysfunction and death through protein deposition, transcription, mitochondrial dysfunction, and direct toxicity of mutant proteins. The precise pathophysiological mechanisms of HD are poorly understood, and there is no effective method to alleviate this disease. Excitingly, the occurrence of emerging technologies and the development of animal model research have brought hope to the treatment of HD.

Although the mechanism of neurodegeneration and dysfunction caused by mHtt is still unknown, an increasing number of studies have confirmed that mitochondrial dysfunction may be an important intermediate factor in the neurotoxicity induced by mHtt [155-157]. SIRT3, which is a major mitochondrial deacetylase, is thought to play an important role in HD. Notably, decreased SIRT3 levels were observed in cells expressing mHtt, while trans-(-)-ε-viniferin could partially restore the expression of SIRT3 and activate AMPK, promoting mitochondrial biogenesis and energy metabolism homeostasis and ultimately protecting cells from mHtt damage [158]. LKB1 is an upstream molecule of AMPK that can regulate AMPK activity. Acetylated LKB1 was significantly increased in Htt-expressing cells, while viniferin could induce SIRT3 to promote the deacetylation of LKB1, thereby increasing the level of phosphorylated AMPK (activated AMPK) and promoting mitochondrial homeostasis [158], suggesting that the SIRT3-LKB1-AMPK pathway plays a crucial role in HD. Intriguingly, SIRT3 overexpression confers neuro-protection in HD by promoting the antioxidant capacity of cells expressing mHtt, leading to enhanced mitochondrial function and balanced dynamics [159]. However, research on the involvement of SIRT3 and its variants in HD is quite limited. A study reported that in an HD model, the mRNA levels of the metabolic regulator SIRT1 were increased in the striatum, cerebral cortex levels of SIRT2 were only increased in the striatum, and SIRT3 was not affected [160]. Notably, Salamon and colleagues assessed the expression patterns of three SIRT3 mRNA isoforms (SIRT3-M1/2/3) in the striatum, cortex and cerebellum by using the N171-82Q transgenic mouse model of HD and found a significant increase in SIRT3-M3 in the striatum and cortex, while transgene in cerebellum led to increased expression of all evaluated subtypes and isoform [161]. These results indicate that in addition to the striatum, which is the most severely affected region, the cerebellum may be another key factor in HD. Protein and enzymatic analyses revealed that SIRT3 was increased in several HD models, including the human HD brain [159]. Overall, SIRT3 exerts neuroprotective effects by maintaining mitochondrial homeostasis by deacetylating factors and regulating oxidative stress. However, different animal models and different methods of analysis and observation have yielded contradictory results regarding the changes in the level of SIRT3 in HD. Whether the expression of SIRT3 is different in different brain regions and cells, whether it is increased or decreased, and whether it has a protective or damaging effect on nerve cells still need to be further studied. Notably, the role of SIRT5, which exhibits weak deacetylase activity, in HD is still poorly understood. The mechanisms by which SIRT4 and SIRT5 regulate mitochondrial function also need to be clarified. Beside these, it is worth mentioning that a potent sirtuin inhibitor known as selisistat especially targeted at SIRT1 while with moderately inhibiting effect on SIRT2/3/6 and no effect on SIRT5 has entered a clinical trial for HD treatment, which exerts positive results in early stage HD patients[162].

### 4.4. Amyotrophic lateral sclerosis

Amyotrophic lateral sclerosis (ALS), also known as motor neuron disease, is a fatal ND that is characterized by the progressive loss of both upper and lower motor neurons, leading to muscle weakness and eventual paralysis [163]. A meta-analysis found that the global standardized incidence of ALS was only 1.68 per 100,000 person-years, but there was also regional and ethnic heterogeneity [164]. Notably, the incidence of ALS increases with age. Statistically, the prevalence of ALS is highest in people aged 60-79 years [165, 166]. Although the pathophysiological mechanisms of ALS have been extensively studied, the disease still faces great challenges from clinical diagnosis to treatment.

Studies on the pathogenesis of ALS have previously focused on SOD1, which encodes Cu/Zn superoxide dismutase; this protein plays a detoxifying role, serving as the main enzyme that catalyzes the transformation of superoxide into hydrogen peroxide and oxygen [167]. However, mutations in SOD1 cause both upper and lower motor neuron loss through a toxic gain-of-function mechanism [168]. The mutation SOD1(G93A) resulted in a significant reduction in mitochondrial length and the accumulation of round fragmented mitochondria, which in turn led to motor neuronal cell death [168]. Intriguingly, SIRT3 and PGC-1α rescued SOD1(G93A)-induced defects by promoting mitochondrial dynamics. In addition, primary astrocytes isolated from mice overexpressing mutant human SOD1 and astrocytes derived from human postmortem ALS spinal cord tissue induced motor neuron death in coculture [169]. Surprisingly, increasing total NAD^+^ and mitochondrial NAD^+^ levels increased oxidative stress resistance and reversed its toxicity to cocultured motor neurons [169]. This is probably achieved by activating sirtuins. A recent study demonstrated that motor neurons in familial and sporadic ALS exhibit similar metabolic deficiencies, characterized by a manganese-specific deficiency in mitochondrial respiration [170]. This correlates with the level of hyperacetylation of mitochondria-associated proteins. Notably, activating SIRT3 reversed the defective metabolic profiles [170]. These results indicate that SIRT3 may play a neuroprotective role in the progression of ALS by promoting mitochondrial dynamics. However, studies of SIRT4 and SIRT5 in ALS are still lacking, and it is not clear whether their expression changes in this disease.

## 5. Mitochondrial sirtuins in fibrosis

Fibrosis is the abnormal deposition of ECM, which can lead to organ dysfunction, disease, and death. A key step in fibrosis is the TGF-β1-mediated shift of fibroblasts into myofibroblasts, which can synthesize ECM [171]. The disease burden caused by fibrosis is large, and there is still no effective treatment to prevent or reverse fibrosis. Mitochondrial sirtuins have been shown to inhibit fibroblast activation and subsequent ECM production, thereby improving organ fibrosis including heart, liver, lung and kidney. With age, SIRT3-KO mice develop tissue fibrosis in multiple organs, including the heart, liver, kidney, and lung [125]. Due to the limited research on SIRT4 and SIRT5, the following sections will focus on SIRT3 (Table 2).

**Table 2:** Summary of the involvement of SIRT3 in inhibiting organ fibrosis.

Models	Organ/tissue	Signaling pathway	Effects	References
Isoprenaline (ISO)-induced myocardial collagen deposition in rat	Heart	SIRT3-TGF-β/Smad	HSY can effectively reduce collagen deposition partly by increasing the SIRT3 expression and inhibiting the protein levels of the components in the TGF-β/Smad pathway.	(Pan et al. 2021)
SIRT3 knockout (KO) mice	Heart	SIRT3-TGF-β/Smad3	Activation of SIRT3 in cardiac fibroblasts both in vivo and in vitro ameliorates cardiac fibrosis and improves cardiac function by inhibiting the TGF-β/Smad3 pathway.	(Chen et al. 2015)
Angiotensin II (Ang-II)-induced cardiac fibrosis in SIRT3 KO mice	Heart	SIRT3-STAT3-NFATc2	SIRT3 overexpression alleviates cardiac fibrosis by deacetylating STAT3 and reducing the expression of NFATc2.	(Guo et al. 2017)
NG2-DsRed-SIRT3 KO mice	Heart	SIRT3-ROS-TGF-β1	Knockout of SIRT3 promotes pericyte-myofibroblast/fibroblast transition which is partly by the mechanisms involving the ROS-TGF-β1 pathway.	(Su et al. 2020)
The human AC16 cell line and SIRT3 knockout mice	Heart	SIRT3-FOS/AP-1	SIRT3 prevents cardiac fibrosis and inflammation via regulating the FOS/AP-1 pathway.	(Palomer et al. 2020)
Spontaneously hypertensive rats	Heart	SIRT3-PARP-1	SIRT3 exhibits anti-fibrosis effect partly by inhibiting the expression of PARP-1.	(Chen, Cheng, et al. 2021)
Hepatic stellate cells (HSC) and Carbon tetrachloride (CCl_4_ )-induced liver fibrosis in rats	Liver	SIRT3-Oxidative Stress	WFA inhibited liver fibrosis through the inhibition of oxidative stress in a SIRT3-dependent manner.	(Gu et al. 2020)
CCl_4_ -induced liver fibrosis in rats	Liver	AMPK-SIRT3	Celastrol attenuates liver fibrosis mainly through inhibition of inflammation by activating AMPK-SIRT3 signaling.	(Wang, Li, et al. 2020)
LX-2 cells (human immortalized HSCs) and CCl_4_ -induced liver fibrosis in mice	Liver	AMPK/SIRT3	HD-16 attenuates CCl4-induced liver inflammation and fibrosis by activating the AMPK/SIRT3 pathway.	(Li et al. 2022)
HSCs and CCl_4_ -induced liver fibrosis in rats	Liver	SIRT3-NF-κB P65	PSG can attenuate inflammation by regulating SIRT3-mediated NF-κB P65 nuclear expression in liver, thus exhibiting anti-fibrotic effect.	(Chen, Gu, et al. 2021)
Iron-induced liver injury	Liver	PPARα-SIRT3-Wnt	PPAR α agonist fenofibrate attenuates iron-induced liver injury in mice by modulating the SIRT3 -Wnt signaling pathway.	(Mandala et al. 2021)
Unilateral ureteral obstruction (UUO) induced fibrosis in SIRT3 KO mice	Kidney	SIRT3-PDHE1α	The deacetylation of PDHE1α by SIRT3 at lysine 385 can prevent renal fibrosis.	(Zhang et al. 2021)
Ischemia-reperfusion-induced acute kidney injury (IR-AKI) models in WT mice and SIRT3 KO mice	Kidney	SIRT3-Mitochondrial function	SIRT3 plays an important role in early-stage fibrosis after IR-AKI by regulating mitochondrial dynamics.	(Cheng et al. 2022)
Ang-II induced fibrosis in SIRT3 KO mice	Kidney	SIRT3- TGFβ1/Iron accumulation /ROS	SIRT3 deficiency sensitized Ang-II-induced renal fibrosis by promoting differentiation of pericytes into fibroblasts, exacerbating iron overload and accelerating NADPH oxidase-derived ROS formation.	(Feng et al. 2020)
Ang-II induced fibrosis in SIRT3 KO mice	Kidney	SIRT3- TGFβ/Smad3	SIRT3 deficiency in endothelial cells stimulates the TGFβ/Smad3-dependent mesenchymal transformations in renal tubular epithelial cells.	(Srivastava et al. 2021)
UUO-induced fibrosis in mice	Kidney	SIRT3/NF-κB/TGF-β1/Smad	HK attenuates UUO-induced renal tubular injury and decreases ECM deposition in mice via SIRT3/NF-κB/TGF-β1/Smad signaling pathway.	(Quan et al. 2020)
Bleomycin induced lung fibrosis in mice	Lung	SIRT3/TGFβ1-SMAD3	Overexpression of SIRT3 attenuates TGFβ1-mediated FMD and significantly reduced the levels of SMAD3.	(Sosulski et al. 2017)
Intratracheal instillation of asbestos or bleomycin to induce lung fibrosis in WT or SIRT3 KO mice. A549 and MLE-12 cell lines	Lung	SIRT3-mtDNA/apoptosis	SIRT3 deficiency promotes lung fibrosis by augmenting alveolar epithelial cell mitochondrial DNA damage and apoptosis.	(Jablonski et al. 2017)
Bleomycin-induced lung fibrosis in SIRT3 KO mice and early-passage human lung fibroblasts (IMR-90)	Lung	SIRT3/TGFβ1	SIRT3 blocks myofibroblast differentiation and pulmonary fibrosis by preventing mitochondrial DNA damage.	(Bindu et al. 2017)
Titanium dioxide or crocidolite asbestos induced lung fibrosis in WT mice or SIRT3^Tg^ mice	Lung	SIRT3	SIRT3 overexpression ameliorates asbestos-induced pulmonary fibrosis, mt-DNA damage, and lung fibrogenic monocyte recruitment.	(Cheresh et al. 2021)

Abbreviations: TGF-β, transforming growth factor-β; HSY, Huangqi Shengmai Yin; STAT3, signal transducer and activator of transcription 3; NFATc2, nuclear factor of activated T cells 2; ROS, reactive oxygen species; AP-1, activator protein-1; PARP-1, ADP-ribose polymerase-1; WFA, withaferin A; AMPK, AMP-activated protein kinase; HD-16, hesperetin derivative 16; PSG, physcion 8-O-β-glucopyranoside; PPARα, peroxisome proliferator-activated receptor α; PDHE1α, pyruvate dehydrogenase E1α; IR-AKI, ischemia-reperfusion-induced AKI; FMD, fibroblast-myofibroblast differentiation.

### 5.1. Cardiac fibrosis

Cardiac fibrosis is the end-stage feature of almost all heart diseases, and it is still an unsolved problem. After the initial injury, cardiac fibroblasts are activated and subsequently differentiate into myofibroblasts, which further promotes ECM renewal and collagen deposition. The accumulation of ECM in the myocardium leads to an increase in the risk of arrhythmia and cardiac injury, which ultimately leads to heart failure. A rat study of isoprenaline-induced myocardial collagen deposition showed that Huangqi Shengmai Yin (HSY) could effectively reduce collagen deposition partly by increasing SIRT3 expression and inhibiting the protein levels of the components in the TGF-β/Smad pathway [172]. This finding suggests that the upregulation of SIRT3 may play a protective role in myocardial fibrosis in rats. Notably, another study on resveratrol in cardiac fibrosis also reached the same conclusion [173]. Resveratrol can activate SIRT3 in cardiac fibroblasts in vivo and in vitro and suppress fibroblast-to-myoblast transformation by inhibiting the TGF-β/Smad3 pathway, ultimately ameliorating cardiac fibrosis and improving cardiac function [173]. In a mouse model of angiotensin II (Ang-II)-induced cardiac fibrosis, SIRT3-KO mice developed more serious cardiac fibrosis than WT controls, while the overexpression of SIRT3 by lentivirus transfection partly reversed these results. Further studies demonstrated that SIRT3 directly binds to and deacetylates signal transducer and activator of transcription 3 (STAT3) to inhibit its activity, which leads to reduced expression of nuclear factor of activated T cells 2 (NFATc2), the downstream factor of STAT3 [174]. In addition, SIRT3 KO accelerated pericyte-myofibroblast/fibroblast transition, promoted Ang-II-induced NADPH oxidase-derived ROS formation and increased the expression of TGF-β1, indicating new mechanisms by which SIRT3 protects against Ang-II-induced cardiac fibrosis [175]. SIRT3 plays a pivotal role in mediating the often-intricate profibrotic effects on cardiac cells by inhibiting the FOS/activator protein-1 (AP-1) pathway [176]. Furthermore, SIRT3 exerts antifibrotic effects partly by maintaining mitochondrial function and inhibiting the expression of poly (ADP-ribose) polymerase-1 (PARP-1) [177]. Although the role of SIRT3 in myocardial fibrosis has been clarified, its effects on upstream and downstream molecules of TGF-β1 still need to be further studied.

### 5.2. Hepatic fibrosis

Hepatic fibrosis often occurs in many chronic liver diseases and is characterized by excessive deposition of ECM protein. Advanced liver fibrosis can lead to cirrhosis, liver failure and portal hypertension, which usually require liver transplantation. Withaferin A (WFA), a bioactive constituent derived from *Withania somnifera*, has been shown to attenuate platelet-derived growth factor BB (PDGF-BB)-induced liver fibrosis in JS1 cells [178]. Notably, SIRT3 depletion attenuated the antifibrogenic effect of WFA, suggesting that WFA inhibited liver fibrosis in a SIRT3-dependent manner. Consistent with the in vitro results, WFA could increase SIRT3 expression and attenuate carbon tetrachloride (CCl_4_ )-induced liver fibrosis in WT mice but not SIRT3-KO mice [178]. However, the signals upstream and downstream of SIRT3 in the mechanism of this antifibrotic effect are unclear. Hepatic stellate cells (HSCs) play an important role in liver fibrosis. Celastrol, a pentacyclic triterpene extracted from *Tripterygium wilfordii*, can effectively improve the activation of hematopoietic stem cells and liver fibrosis by increasing SIRT3 promoter activity and SIRT3 expression [130]. Intriguingly, treatment with compound C (an AMPK inhibitor) or AMPK1α siRNA significantly inhibited SIRT3 expression, suggesting that celastrol attenuates liver fibrosis primarily by activating the AMPK-SIRT3 signaling pathway to inhibit inflammation [130]. Another study showed that AMPK silencing suppressed SIRT3 expression in vitro and in vivo, indicating that AMPK was an upstream target of SIRT3 in liver fibrosis [179]. NF-κB is considered a prototypical transcription factor that can regulate proinflammatory factors, including cytokines, chemokines, and adhesion molecules [180]. An increase in nuclear NF-κB P65 is thought to be an important indicator of NF-κB activation. Chen *et al*. identified that physcion 8-O-β-glucopyranoside (PSG) could attenuate inflammation by regulating SIRT3-mediated NF-κB P65 nuclear expression in the liver, thus exerting an antifibrotic effect [181]. Iron accumulation is usually associated with chronic liver diseases. Notably, a recent study linked iron accumulation to liver fibrosis. Excessive iron accumulation in the liver leads to the downregulation of the PPARα-SIRT3-Wnt signaling pathway, further resulting in fibrosis [182]. Treatment with the PPARα agonist fenofibrate or the known SIRT3 activator honokiol (HK) could reverse these effects and mitigate the progression of fibrosis.

### 5.3. Renal fibrosis

Renal fibrosis is a common feature of chronic kidney disease, which is characterized by the excessive accumulation of ECM in the renal interstitium. Mitochondrial acetylation/deacetylation plays an important role in renal fibrosis metabolism. In the unilateral ureteral obstruction (UUO) mouse model, decreased SIRT3 levels and increased acetylation in mitochondria have been detected [183]. HK prevents renal interstitial fibrosis in UUO mice by activating SIRT3 and reducing mitochondrial acetylation in tubules, which results in the attenuation of tubular atrophy and reduced accumulation of ECM proteins [183]. Notably, SIRT3-KO mice developed obvious mitochondrial acetylation and severe fibrosis after UUO, in contrast to WT mice, suggesting the vital antifibrotic role of SIRT3. Similarly, in an AKI model of ischemia-reperfusion injury, SIRT3 deficiency exacerbated early renal fibrosis [184]. Moreover, SIRT3 deletion significantly exacerbated Ang II-induced fibrosis by promoting the differentiation of pericytes into fibroblasts, exacerbating iron overload and accelerating NADPH oxidase-derived ROS formation [185]. In addition, SIRT3 deficiency in endothelial cells stimulated TGFβ/Smad3-dependent mesenchymal transformations in renal tubular epithelial cells [186]. Treatment with HK attenuated UUO-induced renal tubular injury and decreased ECM deposition in mice via the SIRT3/NF-κB/TGF-β1/Smad signaling pathway [187].

### 5.4. Pulmonary fibrosis

Pulmonary fibrosis is a serious interstitial lung disease that occurs in various clinical environments and is the main cause of mortality [188]. The pathological progression of pulmonary fibrosis caused by abnormal inflammation and repair includes an initial diffuse inflammatory response, interstitial cell proliferation, ECM deposition, and fibrosis [189]. Current evidence has demonstrated that SIRT3 dysregulation promotes pulmonary fibrosis [190]. Oxidative stress contributes to alveolar epithelial cell damage and fibroblast-to-myofibroblast differentiation (FMD), which mediates the pathobiology of pulmonary fibrosis. In mouse models of pulmonary fibrosis and human lung fibroblasts, a reduction in SIRT3 promotes the acetylation (inactivation) of oxidative stress response regulators and FMD, while SIRT3 overexpression weakens TGFβ1-mediated FMD and significantly reduces the levels of SMAD3. Compared with the WT control group, SIRT3 KO mice showed increased fibrosis after intratracheal instillation of bleomycin. In contrast, transgenic mice with systemic SIRT3 overexpression were not affected by bleomycin-induced mtDNA damage and pulmonary fibrosis [191-193]. These results indicate that increasing SIRT3 expression may be a strategy for the treatment of fibrosis.

The role of SIRT4 and SIRT5 in fibrosis is still unclear. However, SIRT4 seems to play a negative role in the process of fibrosis. Recent insights have revealed that SIRT4 inhibits the binding of MnSOD to SIRT3 and increases the acetylation level of MnSOD to reduce its activity, resulting in an increase in ROS accumulation after Ang II stimulation and ultimately promoting hypertrophic growth, fibrosis and cardiac dysfunction [194]. Future research should focus on the enzymatic characteristics of different sirtuins to explore their roles in the pathological process of fibrosis, and the interaction between sirtuins cannot be ignored.

## 6. Relationship between mitochondrial sirtuins and fibrosis in aging

Compelling evidence has demonstrated that mitochondrial sirtuins (SIRT3-5) are involved in aging and age-related diseases [27, 195]. To date, decreased NAD^+^ levels have been detected during aging, which may be a fatal weakness leading to cellular and mitochondrial declines in many age-related pathologies [27]. Recent advances have revealed that the loss of mitochondrial sirtuin function, especially that of SIRT3, is associated with many age-related diseases, including cancer, insulin resistance, heart disease, fibrosis and neurodegeneration [196, 197]. Li *et al*. found that SIRT3 could inhibit p53 activity, leading to growth arrest and aging in the human bladder tumor-derived EJ-p53 cell line [198]. In addition, the absence of SIRT3 attenuated the age-related loss of bone mass in both sexes but had no effect on the skeletons of young mice [199]. SIRT3 deficiency also affects cardiac mitochondrial bioenergetics by promoting the hyperacetylation of optic atrophy 1. SIRT3-KO mice have a shorter life span and more severe age-related heart damage than WT mice; this cardiac pathology is characterized by hypertrophy and fibrosis [200]. Notably, deacetylated optic atrophy 1 ameliorated cardiac reserve capacity and protected the heart against hypertrophy and fibrosis in SIRT3-KO mice. Intriguingly, SIRT4, the sole mitochondrial sirtuin in *Drosophila melanogaster*, can regulate energy homeostasis and longevity by mediating the organismal response to fasting [201].

It is well known that fibrosis usually occurs during aging. GSK3β, one of the signaling kinases that interferes with TGF-β signaling, is a serine/threonine kinase that regulates a wide variety of cellular functions. SIRT3 blocks age-associated tissue fibrosis in mice by deacetylating and activating GSK3β [125]. Notably, the overexpression of SIRT3 cDNA delivered through the airway restored fibrosis regression in elderly mice, which was related to the activation of FOXO3a in fibroblasts, the upregulation of proapoptotic members of the Bcl-2 family, and the recovery of apoptosis susceptibility [202]. In addition, SIRT3 in the aging bladder triggers bladder dysfunction [203]. Compared with that in the young group, SIRT3 expression was decreased in the bladder in the elderly group, leading to an increased level of SIRT3 downstream of the NLRP3 inflammasome, which ultimately resulted in collagen deposition and tissue fibrosis [203]. Taken together, these results indicate that mitochondrial sirtuins, especially SIRT3, play crucial roles in aging and age-related fibrosis.

## 7. Conclusion and prospect

Aging has always been a major, inevitable public health problem that must be addressed. The fibrosis and NDs that accompany aging are also heavy burdens on global public health. Although many cellular and molecular mechanisms of fibrosis and NDs have been revealed, there are still few effective treatment strategies. Fortunately, mitochondrial sirtuins (SIRT3-5) regulate mitochondrial function and cellular survival under various physiological and pathological conditions by modifying multiple mitochondrial proteins. SIRT3-5 may be promising candidates for antifibrosis therapy because they can effectively inhibit fibroblast effector cells and ECM deposition. Additionally, SIRT3-5 exert neuroprotective effects by restraining pathological tau aggregation in AD, repressing dopaminergic neuronal degeneration in PD, and promoting the antioxidant effect in cells expressing mHtt in HD.

Although a large number of studies have reported the beneficial effects of mitochondrial sirtuins in ND and fibrosis, their potential side effects should not be ignored. As previously mentioned, SIRT3 plays a neuroprotective role in a variety of NDs by inhibiting inflammatory responses [62, 65, 66]. Microglial cells are considered immune cells of the central nervous system and play an important role in maintaining the homeostasis of the nervous system. In the pathological process of ND, microglial cells are activated to secrete anti-inflammatory cytokines to reduce nerve damage [204]. However, overactivated microglia secrete large amounts of neurotoxic factors such as ROS and proinflammatory cytokines to damage neurons; this event has become a target for drug development to treat NDs [205, 206]. For example, activation of microglial cells in early AD may promote Aβ clearance and reduce neuronal damage [207]. However, with the chronic activation of microglia, synapses can be phagocytosed in a complement-dependent manner, and the pathological progression of tau can be aggravated by the secretion of a large number of proinflammatory factors [207]. It is worth noting that early overexpression of SIRT3 promotes the migration of microglial cells by upregulating C-X3-C motif chemokine receptor 1 (CX3CR1), which can reduce nerve damage [208]. However, SIRT5 can cause excessive activation of microglial cells and aggravate neuronal injury through desuccinylation of Annexin A1 [209]. This suggests that appropriate targeted therapy must be carried out at the right stage to increase the neuroprotective effect of mitochondrial sirtuins, while blind activation of SIRT3-5 may have the opposite effect. In addition, different effects of SIRT3-5 should be balanced in research on the same disease to obtain maximum benefits.

In conclusion, SIRT3-5 may play different roles in different pathological states or even in different disease models. More research is needed to determine how SIRT3-5 activity can be properly regulated in specific cell types and appropriate stages to maximize the beneficial effects of SIRT3-5 on NDs and fibrosis and avoid unnecessary adverse effects. Furthermore, mitochondrial sirtuins, especially SIRT3, may represent promising therapeutic targets to protect against fibrosis and age-related NDs. Thus, further efforts are warranted to fully elucidate the involvement of SIRT3-5 in the pathologic mechanisms of fibrosis and NDs and to develop novel therapeutic targets.
